# Supersize me: hypotheses on torpor-assisted prehibernation fattening in a boreal bat

**DOI:** 10.1098/rsbl.2024.0291

**Published:** 2024-09-18

**Authors:** Mari A. Fjelldal, Niclas R. Fritzén, Kati M. Suominen, Thomas M. Lilley

**Affiliations:** ^1^ Finnish Museum of Natural History, University of Helsinki, Helsinki, Finland; ^2^ Valsörarna Biological Station, Ostrobothnia Australis, Vasa, Finland

**Keywords:** thermoregulation, phenology, prehibernation fattening, *Eptesicus nilssonii*, torpor, seasonality

## Abstract

Hibernators face an energetic dilemma in the autumn at northern latitudes; while temperatures and food availability decrease, hibernating species need to build fat deposits to survive the winter. During this critical fattening phase, insectivorous boreal bats use torpor to build and conserve their reserves. However, we still know little about temporal variability in torpor use employed by bats during the prehibernation fattening period and how decreasing temperatures and food availability in combination with increasing individual body mass impact this. Here, we present two general hypotheses for explaining temporal torpor patterns observed in a boreal bat (*Eptesicus nilssonii*), in which torpor use (i) facilitates rapid mass gain or (ii) conserves stored body mass. Although temporally separated in our dataset, data on temperature, insect abundance and body mass throughout the prehibernation period indicate that *E. nilssonii* reaches the majority of its overwintering mass before the onset of increasing daytime and night-time torpor use. In combination with low food availability by this point in time, these observations suggest torpor expression may be intended to conserve gained reserves rather than facilitate mass gain. Our study is intended as a first proof of concept for disentangling temporal drivers of torpor in bats during the prehibernation fattening phase.

## 1. Introduction

During winter, when food is scarce or unavailable, many heterothermic species hibernate and thus avoid some of the energetic challenges faced by endotherms [1]. However, winter hibernation generally requires prior accumulation of large energy reserves if a hibernator is to survive until spring [2]. Flying or gliding heterotherms (i.e. birds, bats, and gliders) are restricted in the amount of fat they can carry because of reduced flight manoeuvrability [1] and therefore face trade-offs during the prehibernation fattening period, making this a critical period of their annual cycle. For insectivorous boreal bats, the building of fat deposits coincides with a time of decreasing temperatures and insect abundance [3], adding to their challenge as the autumn progresses. Many hibernating bat species therefore initiate rapid fat accumulation while temperatures and insect abundances are still relatively high [4–8]. Although depositing fat stores early in the season might be more easily facilitated, it prolongs the duration that bats must preserve their large energy stores while elevating mechanical and metabolic power requirements to sustain foraging flights due to increased mass [9]. The timing and magnitude of fat deposition is therefore likely to be carefully managed within species and individuals.

To decrease energetic costs, bats employ torpor across the annual cycle to lower metabolic requirements and thus conserve energy reserves [10]. However, the use of this strategy, i.e. the length and depth of the bouts, is highly impacted by environmental conditions (e.g. [11]), food availability (e.g. [12]) and individual energy reserves (e.g. [13]), due to the dynamic balance of costs and benefits associated with the use of torpor [14]. During the autumn prehibernation fattening period, taking place after the summer breeding season, bats rely on torpor to facilitate fat deposition [3,4,15]. Still, until recently, torpor dynamics in bats during this critical phase have been unstudied. Only recently, torpor patterns in two boreal bat species were described throughout the prehibernation period for the first time [15]. The results revealed strong temporal trends in torpor expression, first increasing daytime torpor and then night-time torpor as the season advanced. However, the decline in temperature throughout the study period did not explain all of the variation observed in the expression of torpor, indicating that there were other temporally variable factors contributing to the strong shifts in strategic management of this behaviour. By increasing torpor use during the daytime before initiating the use of torpor at night, bats likely substantially reduce energetic requirements while still benefiting from foraging activities, thereby optimizing their net energy gain. However, depending on the timing and intensity of the fat-building phase, which can occur within a span of a few weeks [5,6], and the amount of insects available, the true benefit of this temporal and time-restricted specific torpor pattern is unknown.

**Figure 1.**
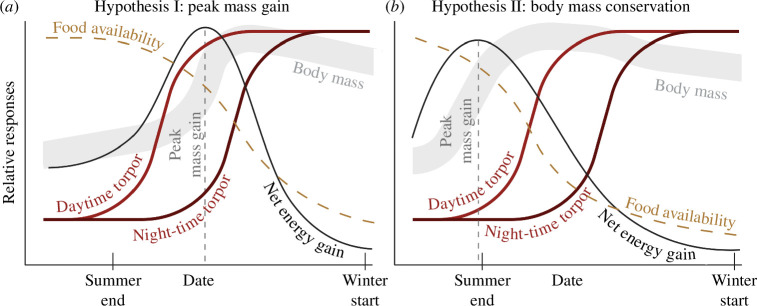
Graphic presentations of our two hypotheses for explaining the temporal torpor patterns observed in the study by Suominen *et al*. [15]. (*a*) Hypothesis I describes the predicted relationships of food availability, net energy gain and body mass expected if the delayed increase in night-time torpor use (while increasing daytime torpor) is intended for maximizing fat deposition. This would require high food availability as net energy gain reflects the energetic pay-off after accounting for costs of foraging flight. (*b*) Hypothesis II assumes that the main body mass increase occurs in the late summer season, and that the increase in daytime and night-time torpor use is expressed to conserve the already accumulated energy deposits as insect food diminishes.

Here, we present two hypotheses to explain both the increase in daytime torpor as well as the following increase in night-time use of torpor and relate these to food availability and increasing body mass during the prehibernation period. First, we hypothesize that peak mass gain is facilitated by maximizing net energy gain through the initiation of increased use of daytime torpor at the onset of decreasing food availability, followed by an increase in the use of night-time torpor after the peak mass gain (figure 1*a*). Alternatively, we hypothesize that body mass conservation after the peak mass gain is facilitated by the increased use of daytime torpor, and further, as foraging opportunities dwindle with further decreasing food availability, the increase in the use of night-time torpor (figure 1*b*). We will examine these hypotheses in a descriptive manner using empirical data on food availability, ambient temperature, and skin temperature and body mass of the medium-sized boreal bat species *Eptesicus nilssonii* (mass 8–13 g, [16]). Because we do not have repeated body mass measurements of individuals with torpor data, and there are temporal discrepancies in data for food availability and mean temperatures, it is not possible to test our hypotheses with statistical models. Therefore, our goal is to provide an initial proof of concept with regards to the two presented hypotheses and encourage further investigation on this critical phase in the yearly cycle of insectivorous bats at high latitudes.

## 2. Methods

**Figure 2.**
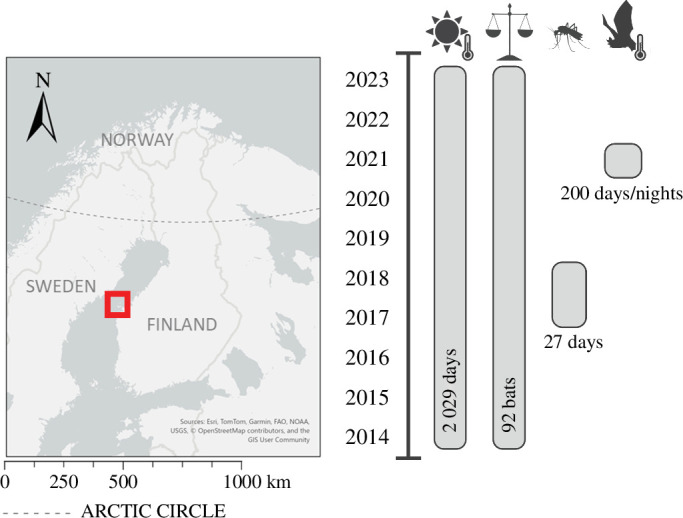
Map indicating location of study area (created with ArcGIS 3.3), and a timeline demonstrating the sampling years of air temperature, body weight measurements, insect collections and skin temperature data of *E. nilssonii*.

The data presented in this study were collected on the Finnish Valsörarna islands (63°27′ N, 21°46′ E) located between Finland and Sweden in the outer archipelago of Kvarken in the Baltic Sea (figure 2). All handling and radio-tagging were carried out under licenses from the ELY Centres (EPOELY/1564/2023 and EPOELY/651/2023 and preceding licenses). All data were processed and analysed in R (v. 4.3.1) and are available in the electronic supplementary material.

### (a) Body mass

The trapping of the bats was conducted within the KvarkenBats project maintained by the Valsörarna Biological Station. Bats were captured with harp-traps, mist-nets or during bat box checks between August and September each year from 2014 to 2023 (see electronic supplementary material, table S1 for yearly captures). Information on sex, age class (adults versus juveniles), forearm length and body mass were recorded for each individual. For the purpose of this study, we only considered the body mass of adult *E. nilssonii* (*N*_obs_ = 92). Initial linear models revealed a strong effect of forearm length on body mass in our data, leading us to construct a body condition index (body mass (g)/forearm length (mm)). We found no effect of time of capture (hours since sunset) and thus excluded time of capture from further analyses. For this study, we were interested in identifying dates indicating changes to the temporal trends in body condition throughout the study period. Therefore, we first performed a Davies’ test (library segmented) to test for the presence of breakpoints in a linear model with body condition index as the response and days since 1 August and sex as explanatory variables. At least one breakpoint was detected (*p* = 0.019) with the best fit suggested around day 29.7. After confirming the presence of breakpoints, we tested for two breakpoints using the segmented function, with initial values set at 20 and 30, respectively, to identify and describe temporal shifts in the body condition index during the prehibernation fattening period.

### (b) Torpor use

The raw data presented here are part of the dataset described in Suominen *et al*. [15]. During one of the trapping years (2021), temperature-sensitive radio transmitters (0.55 g, Telemetrie-Service Dessau, Telemetriesender V4 Temperatur, calibrated by the manufacturer) were attached to the back of captured *E. nilssonii* (*N*_ind_ = 12) using a skin glue (Sauer-Hautkleber). After releasing tagged individuals, pulse frequencies from each transmitter were recorded every minute through an automatic radio-tracking system of 10 RTEU-radio stations on the Valsörarna islands (refer to [17] for details) which picked up the signal of studied bats occupying transitional roosts, such as rock fields, trees and buildings, used in late summer and autumn [15]. We converted these 1 min pulse intervals to skin temperature recordings and calculated 10 min mean skin temperatures. To quantify torpor use, we first calculated a temperature threshold for torpor using the equation presented in [18] and subtracted 2°C due to the expected difference between core and skin temperatures [19], which gave a torpor onset value of 30.0°C for skin temperature. We then applied the methods described in [20] to determine torpor bouts and phases versus euthermic periods, which are identified through the combination of measured skin temperature in relation to the torpor onset value and the change in skin temperature from one measurement to the next. Finally, for each date and individual we calculated daytime (from sunrise to sunset) and night-time (from sunset to sunrise) proportion spent torpid. The proportion spent torpid was calculated by dividing the total time each individual spent in torpor during each day or night with the day or night length.

Due to indications of a temporal shift in torpor use (refer to [15]), we conducted a breakpoint analysis (package and function *segmented*) on the data. The breakpoint analysis was performed on a binomial generalized linear model with the proportion spent torpid as the response and day since 1 August and time of day (day/night) as predictors, to detect a change in the slope. This resulted in a breakpoint estimate 28 days after 1 August (i.e. 29 August). Because the data from before this breakpoint were limited (*N*_days_ = 7 and *N*_nights_ = 5), we only consider data after this breakpoint date. The proportion spent torpid for each day (*N*_days_ = 96) and night (*N*_nights_ = 104) was fitted against date in binomial generalized mixed models, with rainfall and wind speed as additional predictors and individual ID as a random effect. The number of observations per individual ranged from 5 to 28 bat-days (median = 13 days) and from 5 to 30 bat-nights (median = 13 nights).

### (c) Insect abundance

The collection of insects on the Valsörarna islands was initiated as part of a project for mapping the productivity of coastal lagoons and their importance to local ecosystems in the Kvarken area [21]. Insects were collected throughout the summer and autumn in 2017 and 2018 using a single malaise trap on land and two floating eclectors on the water surface (size approx. 0.5 m^2^, at 10 cm depth and at 30–50 cm depth). All traps were emptied on the same day weekly (after 5–9 days). The collected insects were preserved in 70% ethanol and counted based on order or sub-order; for the purpose of this study, we only considered insects belonging to the orders of Ephemeroptera, Lepidoptera (only moths), Nematocera, Neuroptera, Plecoptera and Trichoptera. We merged the counts for all traps for each week and divided the count by the number of days insects had been collected. We only considered samples collected between 1 July until the last collection date each year (1 October and 23 November, respectively). Total counts from these periods were 7729 insects in 2017 and 4234 insects in 2018.

### (d) Ambient temperature conditions

Ambient temperature data throughout summer and autumn for each year from 2014 to 2023 were downloaded with 10 min intervals (*N*_days_ = 2029) from the meteorological station on the Valsörarna islands (Mustasaari Valassaaret), obtained through the Finnish Meteorological Institute.

## 3. Results

**Figure 3.**
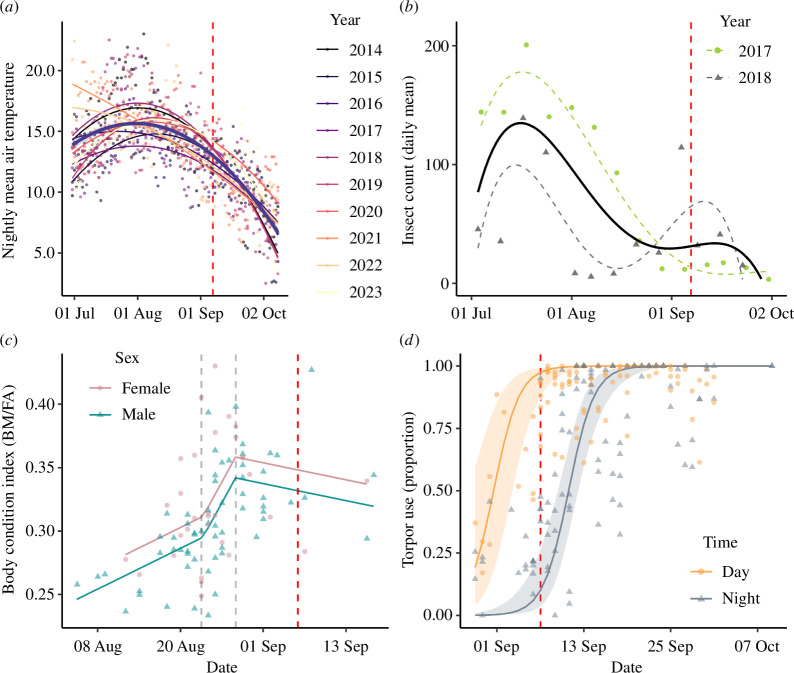
Temporal dynamics across the autumn for data collected during multiple years on the Valsörarna islands (Finland). Red dashed lines mark a common date (7 September) for comparisons between panels. (*a*) Nightly mean air temperatures fitted as quadratic effects against date for each year (2014–2023), with the overall trend across years shown with the thicker dark line. (*b*) Daily mean insect counts for each week during 2 consecutive years (grey and green) and the overall trend for both years (black) fitted as fourth-order polynomials against date. (*c*) Body condition index (mass divided by forearm length) of *E. nilssonii* against date, with males shown in blue and females in pink. Lines correspond to the predicted effects from the fitted breakpoint analysis with two identified break-points (table 1*a*); the first, indicating an increase in body condition, on 24 August, and the second on 29 August, indicating the predicted overall date of the peak body condition (dashed grey lines). (*d*) The predicted logistic effect of torpor use (proportion) by *E. nilssonii* during day (yellow) and night (blue) in autumn, accounting for effects of wind and rain (table 1*b*).

In our high latitude study system (Valsörarna, Finland, 63° N), the empirical data collected during multiple years revealed distinct patterns for the environmental conditions throughout the prehibernation period. Mean ambient temperature (based on data from 2014 to 2023) generally peaked at the end of July before declining steadily throughout August and September (figure 3*a*). However, insect productivity and abundance (based on data from 2017 and 2018) began to decline already in mid-July and reached relatively low levels by the beginning of September, although a single week in September 2018 showed a surge in insect counts (figure 3*b*). Nematocera were by far the most abundant order of insects, accounting for 89% and 99% of the catches in 2017 and 2018, respectively. The order constitutes a major part of the diet of *E. nilssonii* [22]. Therefore, the insect abundance presented can be considered to represent a measure of food availability.

Biometric data from adult *E. nilssonii* collected from 2014 to 2023 revealed strong temporal patterns in the overall body condition index. We identified two breakpoint dates in the data; the first detected at day 23 (i.e. 23 days since 1 August, 24 of August), where the increase in general body condition for males and females intensified after this point in time, until the second breakpoint on day 28 (29 August), marking the overall date of the peak in predicted body condition of our dataset, after which the body condition index began to decline (table 1*a* and figure 3*c*).

**Table 1.** Model results from (*a*) the breakpoint analysis explaining the variation observed in body condition index and (*b*) the logistic model explaining torpor use in autumn.

variable	estimate (s.e.)	*p* value
*a) model: body condition in autumn*		
intercept (female)	0.25 (±0.027)	<0.001
sex (male)	−0.016 (±0.0082)	<0.05
days since 1 August	0.0027 (±0.0013)	<0.05
BP1 (day 23.3)	0.0072 (±0.0074)	NA
BP2 (day 28.0)	−0.011 (±0.0073)	NA
*b) model: torpor use in autumn*		
*ID (random effect*)	4.5 × 10^−9^ (6.7 × 10^−5^)	
intercept (daytime)	−20.52 (±4.21)	<0.001
night-time	−5.84 (±1.22)	<0.001
days since 1 August	0.56 (±0.11)	<0.001
mean wind (m s^−1^)	0.39 (±0.14)	<0.01
total rainfall (mm)	0.50 (±0.16)	<0.01

Torpor patterns for *E. nilssonii* were influenced by date, time of day (night versus day), rain and wind speed (table 1*b*). The date effect predicted a strong increase in daytime torpor use by the end of August, until bats spent entire 24 h periods (day and night) in torpor by early September (figure 3*d*). At this point in time, *E. nilssonii* also begun to increase night-time torpor use, reaching entire nights in torpor by mid-September (figure 3*d*).

Overall, although years during which data were collected differ, the trends of each variable across the prehibernation period indicate that the timing of an increase in daytime torpor use from 29 August (refer Methods) in *E. nilssonii* corresponds to the breakpoint date of when maximum overwintering reserves were generally reached in our dataset, while food availability at this point in time tended to be low. These combined observations support the body mass conservation hypothesis (hypothesis II) rather than the peak mass gain hypothesis (hypothesis I).

## 4. Discussion

Our results suggest the body mass conservation hypothesis (figure 1*b*) is better supported than the peak mass gain hypothesis (figure 1*a*) by the dataset available to us for the study. Although temporally disconnected, the dataset indicates the shift in torpor patterns (approx. 29 August) occurs once *E. nilssonii* has passed the period of peak mass gain (24–29 August), and as the decrease in food availability reaches a plateau. The increase in use of daytime torpor and shift into using night-time torpor could represent a period of opportunistic feeding to maintain body mass dependent on individual state, environmental conditions and momentary food availability. Because bats in this study had reached the majority of their overwintering mass before the onset of shifts in torpor patterns, our dataset is not as supportive of the peak mass gain hypothesis, in which an increase in use of daytime torpor and subsequent shift into using night-time torpor would facilitate reaching maximum body mass.

Previous research on the use of torpor in the autumn has been inconclusive on the contributing drivers, with increasing Julian date being a stronger predictor than decreasing ambient temperatures (although correlated), suggesting that other temporal trends than temperature influence the torpor use observed in these bats during the prehibernation fattening [15]. Here, we propose that the timing of increasing use of daytime torpor in *E. nilssonii*, followed by the increase in night-time torpor, would be best explained by the combined effects of temporal changes to individual state and food availability rather than just decreasing ambient temperature. It is notable that the breakpoint date (29 August) detected for the timing of shifts in torpor use [15] corresponds to the breakpoint date of when maximum overwintering reserves have been reached. Although temporally disconnected, and therefore not available for investigation of direct associations, the data suggest the observed shift in torpor strategies in late August is triggered by individual body condition in *E. nilssonii* reaching a certain level after a rapid peak mass gain period. However, this shift is most likely also influenced by food availability in the environment. Finally, fat synthesis is expected to be restricted in torpid individuals because of metabolic suppression during torpor [23] further supporting our observations of a shift in torpor strategies occurring after the majority of fat reserves have already been stored.

The diet of *E. nilssonii* has been found to consist of Nematocera, a large suborder of Diptera containing mosquitoes and midges [22], in particular. Incidentally, Nematocera accounted for the majority of insects collected in this study and therefore, the temporal trend in insect decline described here is highly representative of the food availability for *E. nilssonii*, aligning with the predictions of the body mass conservation hypothesis (figure 1*b*). The stochasticity of food availability within and across years increases with an increase in latitude due to overall lower insect abundances [24]. The unpredictable nature of food availability can contribute to a pattern in which maximum body mass is reached in advance of the ultimate decline in insect abundance, and switch to body mass conservation during a period when food availability is uncertain. Similarly, flexible use of torpor to assist fat storage despite availability of food has also been observed in marsupials [25].

Although not evident in our system, the first hypothesis can also hold true for bats, depending on their feeding strategy. The early and rapid fattening, as observed here, appears to be mainly observed in ‘hawker’ bat species foraging on small aerial insects that decline rapidly in autumn, while ‘gleaner’ species, hunting terrestrial arthropod prey from the ground or vegetation surfaces, have food available for longer and can delay the onset or reduce the intensity of the lipogenesis [26]. In the study by [27], species such as *Myotis nattereri* and *M. bechsteinii,* with non-specialist diets composed of both aerial and terrestrial arthropods, appear to strongly delay the onset of prehibernation fattening or decrease their rate of mass gain. On the other hand, *M. daubentonii*, a hawker bat species hunting mainly aquatic insects, was observed to increase its body mass earlier and/or more rapidly than any of the gleaning species. These observations are similar to those presented by [26] and support our overall hypothesis of food availability being one of the strongest drivers of species-specific prehibernation fattening strategies in insectivorous bats. We can, therefore, expect torpor patterns in autumn to also differ between species as a response to variability in expected foraging success and individual body condition throughout autumn.

Finally, we highlight the need for empirical studies measuring the collective temporal trends of torpor use, food availability and body mass gain in bats during the critical prehibernation phase to better understand interactions between environmental and individual conditions on thermoregulatory strategies. We see this initial foray in interpreting observed autumn torpor expressions in *E. nilssonii,* by investigating general temporal trends within the study system, as a basis for further studies that can provide a more conclusive understanding on how hibernators can cope in a rapidly changing environment.

## Data Availability

All data used in this study are included as electronic supplementary material [28].

## References

[1] Geiser F. 2021 Ecological physiology of daily torpor and hibernation. Berlin, Germany: Springer.

[2] Humphries MM, Thomas DW, Kramer DL. 2003 The role of energy availability in mammalian hibernation: a cost–benefit approach. Physiol. Biochem. Zool. **76**, 165–179. (10.1086/367950) 12794670

[3] Speakman JR, Rowland A. 1999 Preparing for inactivity: how insectivorous bats deposit a fat store for hibernation. Proc. Nutr. Soc. **58**, 123–131. (10.1079/pns19990017) 10343349

[4] McGuire LP, Fenton MB, Guglielmo CG. 2009 Effect of age on energy storage during prehibernation swarming in little brown bats (Myotis lucifugus). Can. J. Zool. **87**, 515–519. (10.1139/Z09-041)

[5] Kronfeld-Schor N, Richardson C, Silvia BA, Kunz TH, Widmaier EP. 2000 Dissociation of leptin secretion and adiposity during prehibernatory fattening in little brown bats. Am. J. Physiol. Regul. Integr. Comp. Physiol. **279**, R1277–R1281. (10.1152/ajpregu.2000.279.4.R1277) 11003993

[6] Kunz TH, Wrazen JA, Burnett CD. 1998 Changes in body mass and fat reserves in pre-hibernating little brown bats (Myotis lucifugus). Écoscience. **5**, 8–17. (10.1080/11956860.1998.11682443)

[7] Suba J, Vintulis V, Petersons G. 2010 Body weight provides insights into the feeding strategy of swarming bats. Hystrix. Ital. J. Mammal. **22**, 179–187. (10.4404/hystrix-22.1-4516)

[8] Fraser EE, McGuire LP. 2023 Prehibernation swarming in temperate bats: a critical transition between summer activity and hibernation. Can. J. Zool. **101**, 408–422. (10.1139/cjz-2022-0129)

[9] Hughes PM, Rayner JMV. 1991 Addition of artificial loads to long-eared bats Plecotus auritus: handicapping flight performance. J. Exp. Biol. **161**, 285–298. (10.1242/jeb.161.1.285)

[10] Stawski C, Willis CKR, Geiser F. 2014 The importance of temporal heterothermy in bats. J. Zool. **292**, 86–100. (10.1111/jzo.12105)

[11] Wojciechowski MS, Jefimow M, Tęgowska E. 2007 Environmental conditions, rather than season, determine torpor use and temperature selection in large mouse-eared bats (Myotis myotis). Comp. Biochem. Physiol. A Mol. Integr. Physiol. **147**, 828–840. (10.1016/j.cbpa.2006.06.039) 16891137

[12] Coburn DK, Geiser F. 1998 Seasonal changes in energetics and torpor patterns in the subtropical blossom-bat Syconycteris australis (Megachiroptera). Oecologia **113**, 467–473. (10.1007/s004420050399) 28308026

[13] Fjelldal MA, Muller AS, Ratikainen II, Stawski C, Wright J. 2023 The small-bat-in-summer paradigm: energetics and adaptive behavioural routines of bats investigated through a stochastic dynamic model. J. Anim. Ecol. **92**, 2078–2093. (10.1111/1365-2656.13999) 37661664

[14] Boyles JG, Johnson JS, Blomberg A, Lilley TM. 2020 Optimal hibernation theory. Mamm. Rev. **50**, 91–100. (10.1111/mam.12181)

[15] Suominen KM, Fritzén NR, Fjelldal MA, Blomberg AS, Viljamaa MJK, Lilley TM. 2024 Thermoregulation and diurnal roost selection of boreal bats during pre-hibernation period. bioRxiv. (10.1101/2024.05.22.595441)

[16] Suominen, KM, Kotila, M, Blomberg, AS, Pihlström, H, Ilyukha, V, Lilley, TM. 2020 Northern bat *Eptesicus nilssonii* (Keyserling and Blasius, 1839). In Handbook of the mammals of Europe, pp. 1–27. Cham, Switzerland: Springer. (10.1007/978-3-319-65038-8_45-1)

[17] Gottwald J, Zeidler R, Friess N, Ludwig M, Reudenbach C, Nauss T. 2019 Introduction of an automatic and open‐source radio‐tracking system for small animals. Methods Ecol. Evol. **10**, 2163–2172. (10.1111/2041-210X.13294)

[18] Willis CKR. 2007 An energy-based body temperature threshold between torpor and normothermia for small mammals. Physiol. Biochem. Zool. **80**, 643–651. (10.1086/521085) 17910000

[19] Audet D, Thomas DW. 1996 Evaluation of the accuracy of body temperature measurement using external radio transmitters. Can. J. Zool. **74**, 1778–1781. (10.1139/z96-196)

[20] Fjelldal MA, Stawski C, Sørås R, Wright J. 2023 Determining the different phases of torpor from skin- or body temperature data in heterotherms. J. Therm. Biol. **111**, 103396. (10.1016/j.jtherbio.2022.103396) 36585072

[21] Schneider M, Fritzén NR. 2020 Flador och deras insektproduktion—betydelsen för lokala och migrerande fladdermöss i Kvarken. Delrapport inom Interreg Botnia Atlantica projekt Kvarken Flada. See https://files.builder.misssite.com/98/25/9825f3aa-e3e0-4661-b43b-dab5846d85c1.pdf.

[22] Vesterinen EJ, Puisto AIE, Blomberg AS, Lilley TM. 2018 Table for five, please: dietary partitioning in boreal bats. Ecol. Evol. **8**, 10914–10937. (10.1002/ece3.4559) 30519417 PMC6262732

[23] Geiser F. 1988 Reduction of metabolism during hibernation and daily torpor in mammals and birds: temperature effect or physiological inhibition? J. Comp. Physiol. B. **158**, 25–37. (10.1007/BF00692726) 3385059

[24] Pöyry J, Leinonen R, Söderman G, Nieminen M, Heikkinen RK, Carter TR. 2011 Climate-induced increase of moth multivoltinism in boreal regions. Glob. Ecol. Biogeogr. **20**, 289–298. (10.1111/j.1466-8238.2010.00597.x)

[25] Geiser F, Masters P. 1994 Torpor in relation to reproduction in the mulgara, Dasycercus cristicauda (Dasyuridae: Marsupialia). J. Therm. Biol. **19**, 33–40. (10.1016/0306-4565(94)90007-8)

[26] Reiter A, Benda P, HoffmannovÁ A, Andreas M. 2010 Project: swarming bats in Ledové sluje. In A tribute to bats (eds I Horácek, M Uhrin), pp. 127–138. Kostelec nad Černými lesy, Czech Republic: The Publishing House Lesnická práce, sro.

[27] Ignaczak M, Postawa T, Lesiński G, Gottfried I. 2019 The role of autumnal swarming behaviour and ambient air temperature in the variation of body mass in temperate bat species. Hystrix **30**, 65. (10.4404/hystrix-00104-2018)

[28] Fjelldal MA, Fritzén NR, Suominen KM, Lilley T. 2024 Data from: Supersize me: hypotheses on torpor-assisted prehibernation fattening in a boreal bat. Figshare. (10.6084/m9.figshare.c.7440152)39288816

